# Volatile organic compounds produced by the phytopathogenic bacterium *Xanthomonas campestris* pv. *vesicatoria* 85-10

**DOI:** 10.3762/bjoc.8.65

**Published:** 2012-04-17

**Authors:** Teresa Weise, Marco Kai, Anja Gummesson, Armin Troeger, Stephan von Reuß, Silvia Piepenborn, Francine Kosterka, Martin Sklorz, Ralf Zimmermann, Wittko Francke, Birgit Piechulla

**Affiliations:** 1 University of Rostock, Institute of Biological Sciences, Albert-Einstein-Str. 3, 18059 Rostock, Germany; 2 present address: Max-Planck Institute for Chemical Ecology, Hans-Knoell-Str. 8, 07745 Jena, Germany; 3 Joint Mass Spectrometry Centre of the University of Rostock, Chair of Analytical Chemistry, Albert-Einstein-Str. 1, 18059 Rostock, Germany and the Cooperation group „Comprehensive Molecular Profiling“, Helmholtz Zentrum München, Ingolstädter Landstraße 1, 85764 Oberschleißheim, Germany; 4 University of Hamburg, Institute of Organic Chemistry, Martin-Luther-King-Platz 6, 20146 Hamburg, Germany; 5 present address: Boyce Thompson Institute, Cornell University, 1 Tower Road, Ithaca, NY, 14853, USA

**Keywords:** *Aspergillus nidulans*, *Fusarium solani*, growth inhibition and promotion, methylketones, 10-methylundecan-2-one, *Rhizoctonia solani*, volatile organic compound (VOC), *Xanthomonas campestris* pv. *vesicatoria*

## Abstract

*Xanthomonas campestris* is a phytopathogenic bacterium and causes many diseases of agricultural relevance. Volatiles were shown to be important in inter- and intraorganismic attraction and defense reactions. Recently it became apparent that also bacteria emit a plethora of volatiles, which influence other organisms such as invertebrates, plants and fungi. As a first step to study volatile-based bacterial–plant interactions, the emission profile of *Xanthomonas c.* pv. *vesicatoria* 85-10 was determined by using GC/MS and PTR–MS techniques. More than 50 compounds were emitted by this species, the majority comprising ketones and methylketones. The structure of the dominant compound, 10-methylundecan-2-one, was assigned on the basis of its analytical data, obtained by GC/MS and verified by comparison of these data with those of a synthetic reference sample. Application of commercially available decan-2-one, undecan-2-one, dodecan-2-one, and the newly synthesized 10-methylundecan-2-one in bi-partite Petri dish bioassays revealed growth promotions in low quantities (0.01 to 10 μmol), whereas decan-2-one at 100 μmol caused growth inhibitions of the fungus *Rhizoctonia solani*. Volatile emission profiles of the bacteria were different for growth on media (nutrient broth) with or without glucose.

## Introduction

Plant surfaces are inhabited by diverse and complex communities of microorganisms, although these habitats may be hostile environments, e.g. [1–6]. Bacteria are the most dominant inhabitants, e.g., more than 10^7^ cells per cm^2^ of leaf surface are present, but also filamentous fungi and yeasts are found [7–8]. One of the most abundant bacterial genera in the phyllosphere is *Xanthomonas*; *X. campestris* is the dominant species, and at least 141 pathovars invasive against several plant species are known, including many of agricultural relevance [9]. Prominent and widespread diseases caused by *Xanthomonas* species include diseases such as bacterial spot on peppers and tomatoes, citrus canker, and bacterial blight disease in rice [9–10]. Unravelling the mechanisms for phytopathogenicity and virulence of *X. campestris* resulted in the identification of a type-three secretion system through which bacterial effector proteins enter the plant cells to interfere with cellular processes, to the benefit of the bacterium [11]. However, some plants react to the effector proteins by local cell death, a hypersensitive response, and are able to escape bacterial spreading and invasion. Apart from these cell-to-cell-based interactions, additional modes of action of *X. campestris* may influence growth of the plant directly or indirectly. Although antagonistic features of *Xanthomonas* spp. against fungi have been only sparsely studied, it is known that *Xanthomonas* competes with fungi [3]. The bacteria take up nutrients more rapidly and in larger amounts as compared to the germ tubes of fungi. This could be advantageous for *Xanthomonas* during competition for nutrients. Furthermore, some antagonistic activity of two *Xanthomonas* isolates against *Verticillium dahliae* was documented [12]. Since only a little or no production of lytic enzymes or siderophores could be observed, other mechanisms must exist that promote bacterial growth versus fungal growth. It was shown that 11-methyldodec-2*Z*-enoic acid, known as quorum-sensing signal, produced by *X. campestris* pv. *campestris* repressed hyphal development in *Candida albicans* [13], and Hogan et al. [14] concluded that either the bacteria modulate the fungal behavior or that *C. albicans* (or related fungi) responded to the presence of antagonistic bacteria in such a way that it was advantageous for the bacteria.

Volatiles were also shown to support and facilitate cross-kingdom interactions, such as plant–insect communications as bi- and tritrophic attractions and defenses [15]. For example, volatiles emitted by vegetative plant tissues suppress the growth of microorganisms, both in the case of bacteria and fungi [16–19]. Recently, a new field of interest emerged, when it became apparent that also bacteria emit a plethora of volatiles, which may influence other organisms, such as invertebrates, plants and fungi [20–21]. There is increasing information about those volatiles emitted by bacteria, especially rhizobacteria, e.g., [22–25], which influence the growth of fungi [22–23,26–27]. In most cases the bacterial volatiles showed inhibitory activities (reviewed in [20]). We, therefore, hypothesized that volatile compounds emitted by *Xanthomonas campestris* pv. *vesicatoria* 85-10 may also play a role as antagonistic weapons against competitive fungi. As a first approach in our investigations, we analysed the profiles of its volatiles. Only two volatile organic compounds released by *X. campestris* have so far been described: 11-methyldodec-2*Z*-enoic acid [13] and γ-butyrolactone [28]. To extend this preliminary information we carried out GC/MS and proton transfer reaction (PTR)–MS analyses with *X. c.* pv. *vesicatoria* 85-10. In addition, we aimed at structure elucidation and on temporal variation of profiles of volatiles produced upon growth on different media.

## Results and Discussion

### Effect of *Xanthomonas campestris* pv. *vesicatoria* volatiles on fungal growth

**Figure 1:**
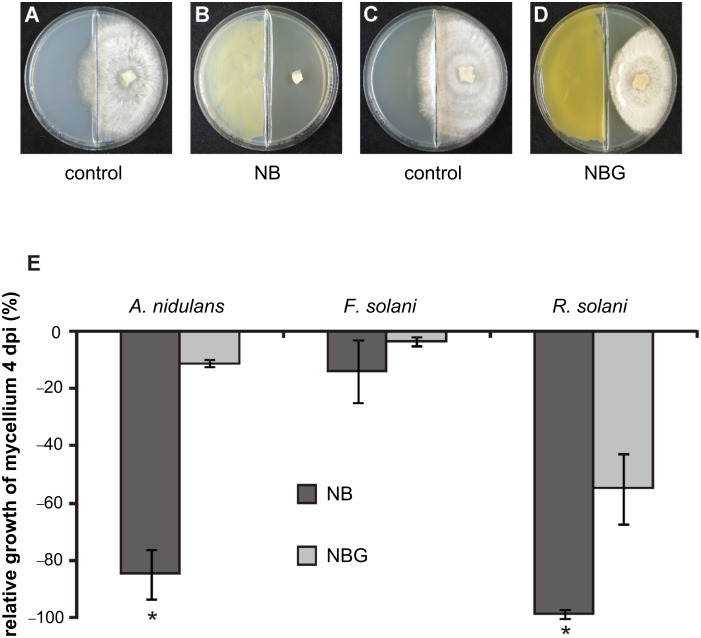
Cocultivation of *Xanthomonas campestris* pv. *vesicatoria* 85-10 with three fungi on different media. (A) Control experiment: Cultivation of *Rhizoctonia solani* on NB (day 4). (B) Cocultivation of *X. c.* pv. *vesicatoria* 85-10 on NB with *Rhizoctonia solani* (day 4). (C) Control experiment: Cultivation of *Rhizoctonia solani* on NBG (day 4). (D) Cocultivation of *X. c.* pv. *vesicatoria* 85-10 on NBG with *Rhizoctonia solani* (day 4). (E) Quantification of the inhibition of fungal mycelium growth during cocultivation with *X. c.* pv. *vesicatoria* 85-10, either grown on NB (black column) or NBG (grey column). Fungi: *Aspergillus nidulans, Fusarium solani* and *Rhizoctonia solani*. (4 dpi: four days after inoculation; NBG: nutrient broth (NB) II agar plus 1.1% glucose; *: growth significantly different compared to the control, p < 0.01 according to t-test).

So far neglected in former investigations was the possibility that volatiles emitted from *X. c.* pv. *vesicatoria* 85-10 may influence plant growth directly or indirectly, e.g., through fungi. As a first step to investigate whether *Xanthomonas* volatiles effect other organisms, three fungi, *Aspergillus nidulans*, *Fusarium solani* and *Rhizoctonia solani,* were cocultivated with *X. c.* pv. *vesicatoria* 85-10 in compartmentalized Petri dishes (Figure 1). This ensures that only volatiles can diffuse between the compartments to act on the fungal test organisms. Since it is known that growth media influence the pattern of bacterial volatiles [29], nutrient broth with and without the addition of glucose, NBG and NB, respectively, were investigated. Figure 1A–D demonstrates the effects of *Xanthomonas* volatiles on *R. solani* in such a culture system. In the absence of bacteria the fungus exhibits circular mycelium growth (control, Figure 1A and Figure 1C), while growth was retarded when *X. c.* pv. *vesicatoria* 85-10 was growing in the other compartment (Figure 1B and Figure 1D). Growth of *R. solani* was more inhibited when *X. c.* pv. *vesicatoria* 85-10 grew on NB compared to less inhibition when it was grown on NBG (98% vs 55%, Figure 1B and Figure 1D, respectively). The same tendency was observed for the growth of *A. nidulans* and *F. solani*. When *X. c.* pv. *vesicatoria* 85-10 grew on NB, *A. nidulans* was inhibited by 85% and *F. solani* by 14%, while the inhibition of both fungi on NBG was only 11% and 3.5%, respectively. The inhibitory potential of *X. c.* pv. *vesicatoria* 85-10 volatiles on fungi was less pronounced when the bacteria grew on glucose-containing media (Figure 1E), indicating that growth suppression depended on the supplied nutrient source. Fiddaman and Rossall [29] described similar results, since the addition of D-glucose but not L-glucose lead to the formation of inhibitory volatiles by *Bacillus subtilis*. The milder inhibitory potential of *X. c.* pv. *vesicatoria* 85-10 growing on NBG, may be explained by the suggestions that (i) inhibitory volatiles were produced in higher amounts when grown on NB medium, (ii) inhibitory volatiles were only produced in the peptone-rich NB medium, (iii) glucose suppressed the production of inhibitory volatiles, for example through catabolite repression, or (iv) the emission of inhibitory volatiles was delayed.

Interestingly, *Fusarium solani* was only weakly influenced by the volatiles of *X. c.* pv. *vesicatoria* 85-10, indicating species-specific reactions. Similar results were obtained when ten different rhizobacteria were tested with various fungi [27]. Most of the tested fungi, including *R. solani*, *Verticillium dahlia*e, *Paecilomyces carneus* and *Sclerotinia sclerotiorum* were strongly inhibited by the rhizobacteria (*Serratia* spp., *Pseudomonas* spp., *Stenotrophomonas* spp.), and only *F. solani* appeared to be resistant against the bacterial volatiles. Also *Muscodor albus* volatiles only partially influence the growth of *F. solani*, while other fungi are much more severely inhibited [30]. Bacterial volatiles may also directly influence plant growth. *Arabidopsis thaliana* and *Physcomitrella patens* were exposed to mixtures of volatiles emitted by various bacteria. Depending on the bacterial species or experimental setup, either promotion or inhibition of plant growth was observed [25,27,31–35]. Individual compounds such as acetoin and 2,3-butanediol acted as plant-growth-promoting agents [31], while dimethyl disulfide and 2-phenylethanol inhibited plant growth [34,36]. The inhibitory potential of volatiles of different *Xanthomonas campestris* species/isolates on *Caenorhabditis elegans* and on bacteria was also shown [20,37].

### Volatile emissions of *Xanthomonas campestris* pv. *vesicatoria* 85-10

#### GC/MS analysis of volatiles released by *X. c.* pv. vesicatoria 85-10

**Figure 2:**
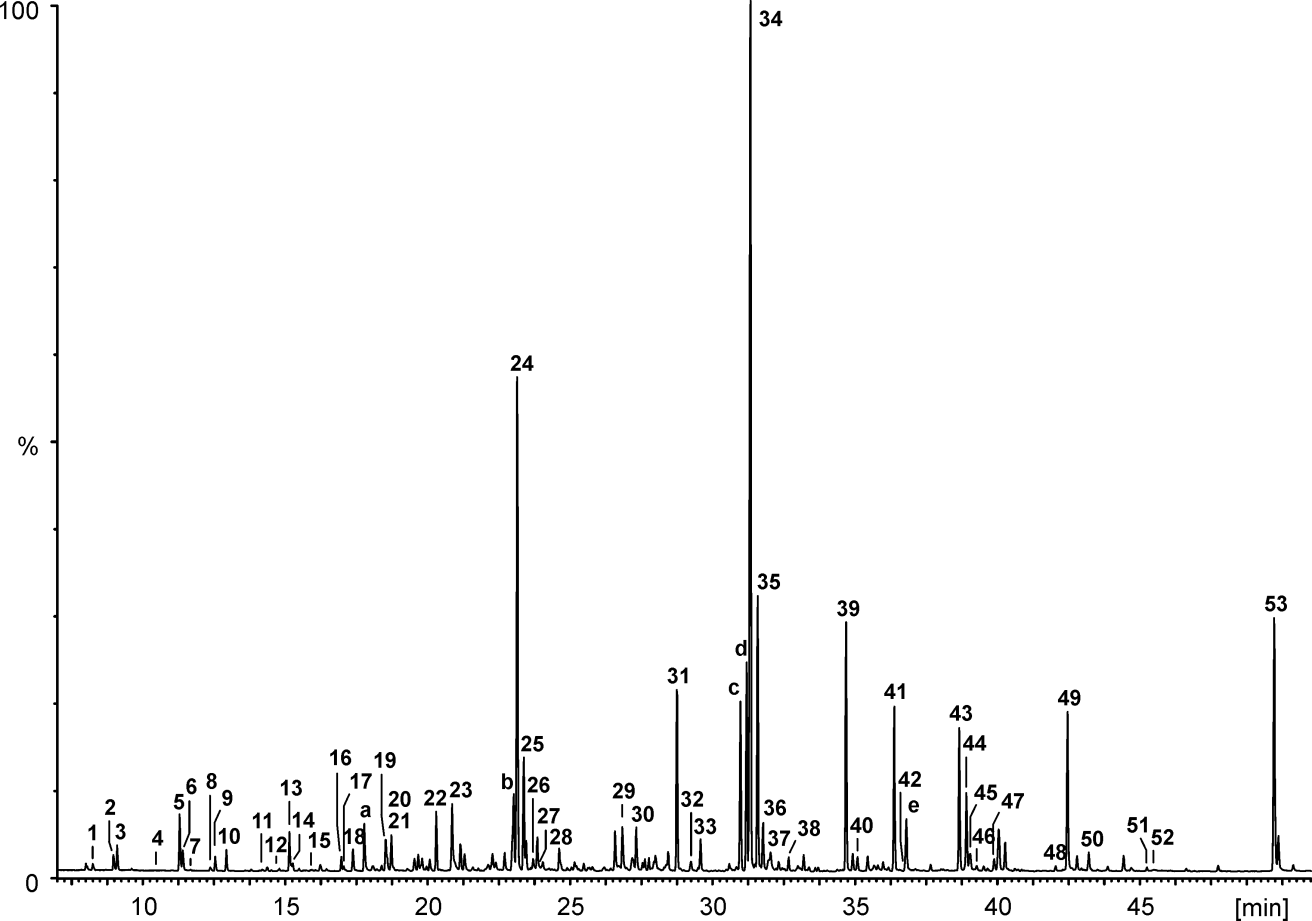
GC/MS-chromatogram (total ion current) of the headspace of *X. c.* pv. *vesicatoria* 85-10 grown in 10 L liquid nutrient broth without glucose. Compound labels are the same throughout this paper.

**Figure 3:**
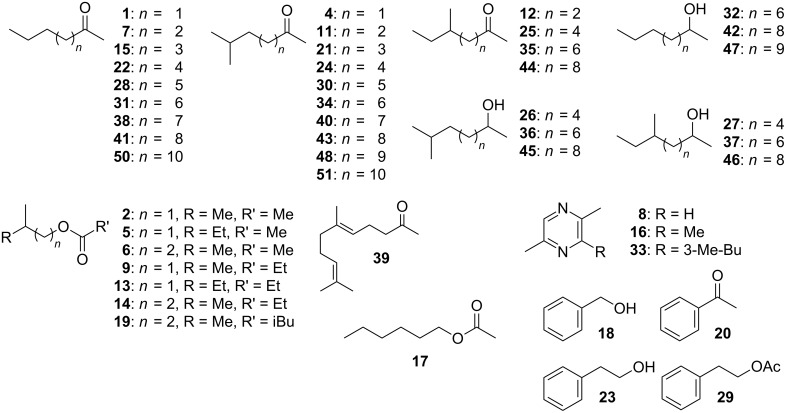
Structures of compounds emitted by *Xanthomonas campestris* pv. *vesicatoria* 85-10. Compound labels are the same throughout this paper.

**Scheme 1:**
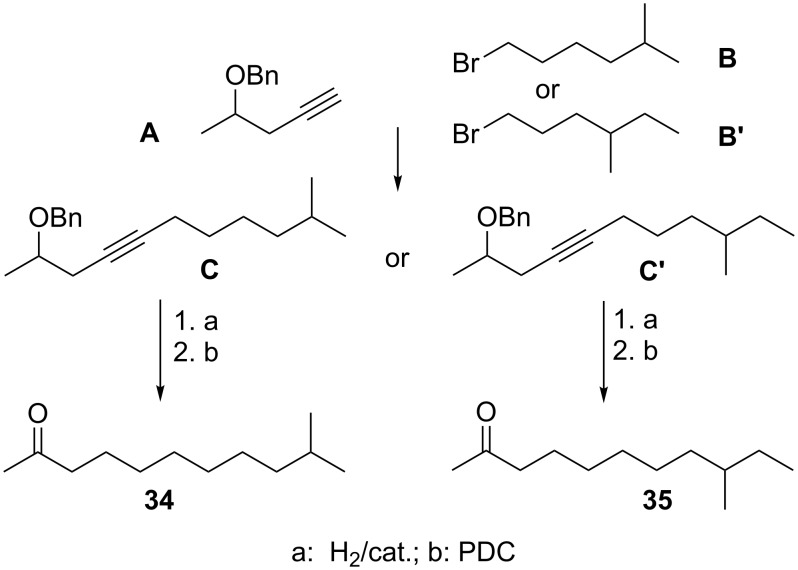
Synthesis of 10-methylundecan-2-one (**34**) and 9-methylundecan-2-one (**35**).

The volatiles emitted by *X. c.* pv. *vesicatoria* 85-10 showed strong effects on the tested fungi (Figure 1). Therefore, it was interesting to carry out investigations on the qualitative and quantitative profiles of volatiles produced by *X. c.* pv. *vesicatoria* 85-10. Samples of volatiles were obtained by growing the bacteria in 10 L of liquid medium and trapping the volatiles on SuperQ. Upon application of GC/MS, more than 50 compounds were identified among a large number of components present in only very small amounts (Figure 2, Figure 3, Table 1). Contaminants (see Table 1) are marked in Figure 2. The scent released by *X. c.* pv. *vesicatoria* 85-10 was shown to be particularly rich in ketones and corresponding alcohols. In addition to straight-chain methylketones from hexan-2-one (compound **1** in Figure 3 and Table 1) to pentadecan-2-one (**50**), several branched-chain methylketones could be identified (Figure 3, Table 1). The volatiles were found to be largely dominated by a substance (Figure 2, compound **34**) showing a mass spectrum that was very similar to that of dodecan-2-one. A slightly later-eluting minor component furnished an almost identical mass spectrum. As both compounds eluted between undecan-2-one (**31**) and dodecan-2-one (**38**) (small amounts of which were found to be present among the natural volatiles), retention indices with methylketones as reference standards were calculated. Corresponding data published for methyl-branched hydrocarbons and increment calculations strongly suggested *iso*-branching for the main compound and *anteiso*-branching for the later-eluting isomer [38–40]. In fact, mass spectra and retention times (co-injection) of 10-methylundecan-2-one (**34**) and 9-methylundecan-2-one (**35**) synthesized in our lab (Scheme 1) matched those of the respective natural products. Small amounts of the corresponding alcohols, 10-methylundecan-2-ol (**36**) and 9-methylundecan-2-ol (**37**) (stereochemistry not determined) were also detected by GC/MS and co-injection. As the headspace volatiles contained a continuous row of homologous straight-chain methylketones, identification of additional compounds with similar structures was largely facilitated: the unbranched ketones were found to be always accompanied by the *iso*-branched isomers and in some cases also by the *anteiso*-branched ones. Corresponding methylcarbinols could also be detected. The position of the double bond in a pentadecen-2-one (**49**), a member of the identified suite of methylketones, remained unassigned.

**Table 1:** GC/MS analysis of Super-Q trapped volatiles emitted by *Xanthomonas campestris* pv. *vesicatoria* 85-10 grown in 10 L nutrient broth without glucose. Compound labels are the same throughout this paper.

compound	*t*_R_ [min]	retention index I	rel. area [%]	compound

**1**	8.23	778	0.7	hexan-2-one
**2**	8.96	795	1.6	2-methylpropyl acetate
**3**	9.10	798	2.5	*n*-octane
**4**	10.50	830	tr	5-methylhexan-2-one
**5**	11.28	848	5.4	2-methylbutyl acetate
**6**	11.40	851	2.4	3-methylbutyl acetate
**7**	11.67	857	0.2	heptan-2-one
**8**	12.36	873	0.4	2,5-dimethylpyrazine
**9**	12.53	877	1.4	2-methylpropyl propionate
**10**	12.92	886	2.0	*n*-nonane
**11**	14.36	919	0.5	6-methylheptan-2-one
**12**	14.78	929	tr	5-methylheptan-2-one
**13**	15.14	938	3.9	2-methylbutyl propionate
**14**	15.26	940	0.7	3-methylbutyl propionate
**15**	15.88	955	tr	octan-2-one
**16**	16.22	963	0.9	2,3,5-trimethylpyrazine
**17**	16.97	981	1.3	hexyl acetate
**18**	17.37	990	2.5	benzylalcohol
**19**	18.51	1018	4.0	3-methylbutyl 3-methylbutyrate
**20**	18.71	1022	3.7	acetophenone
**21**	18.71	1022	tr	7-methyloctan-2-one
**22**	20.29	1061	5.6	nonan-2-one
**23**	20.85	1074	8.4	2-phenylethanol
**24**	23.13	1131	52.8	8-methylnonan-2-one
**25**	23.37	1137	11.6	7-methylnonan-2-one
**26**	23.69	1145	1.4	8-methylnonan-2-ol
**27**	23.99	1152	tr	7-methylnonan-2-ol
**28**	24.60	1168	2.9	decan-2-one
**29**	26.82	1224	5.5	2-phenylethyl acetate
**30**	27.31	1237	4.9	9-methyldecan-2-one
**31**	28.73	1274	19.7	undecan-2-one
**32**	29.23	1287	1.4	undecan-2-ol
**33**	29.56	1296	3.7	3,6-dimethyl-2-(3-methylbutyl)pyrazine
**34**	31.32	1344	100.0	10-methylundecan-2-one
**35**	31.58	1351	28.3	9-methylundecan-2-one
**36**	31.76	1356	4.9	10-methylundecan-2-ol
**37**	32.02	1363	4.0	9-methyundecan-2-ol
**38**	32.66	1380	1.4	dodecan-2-one
**39**	34.68	1437	25.0	geranylacetone
**40**	35.08	1448	1.7	11-methyldodecan-2-one
**41**	36.37	1485	17.5	tridecan-2-one
**42**	36.76	1496	tr	tridecan-2-ol
**43**	38.64	1552	15.7	12-methyltridecan-2-one
**44**	38.89	1559	8.8	11-methyltridecan-2-one
**45**	39.03	1563	3.0	12-methyltridecan-2-ol
**46**	39.27	1570	0.7	11-methyltridecan-2-ol
**47**	39.87	1589	1.4	tetradecan-2-ol
**48**	42.02	1655	0.6	13-methyltetradecan-2-one
**49**	42.45	1668	17.7	pentadecen-2-one
**50**	43.19	1691	2.1	pentadecan-2-one
**51**	45.24	1757	0.5	14-methylpentadecan-2-one
**52**	45.48	1765	0.4	13-methylpentadecan-2-one
**53**	49.69	1907	29.8	terpenoid?
**contaminants, also in blank**				
**a**	17.76	1000	5.1	2-ethylhexanol
**b**	23.01	1128	8.8	2-ethylhexyl acetate
**c**	30.97	1334	17.8	hydrocarbon
**d**	31.19	1340	20.5	hydrocarbon
**e**	36.79	1497	6.7	2,4-bis-*tert*-butylphenol

Some of the identified ketones released by *X. c.* pv. *vesicatoria* 85-10 were known to be emitted by other bacteria, e.g., undecan-2-one (**31**) was emitted from *Serratia* sp. 2675*, Pseudomanas fluorescens*, *P*. *chlororaphis*, *P*. *aurantiaca* and *P*. *corrugata* [22,41]. Dodecan-2-one (**38**) was found among the volatiles of *Serratia* strains [41], and decan-2-one (**28**) was released by an epilithic cyanobacterial biofilm of *Rivularia* sp. and *Calothrix parietina* [42]. The main component among the volatile compounds released by *X. c.* pv. *vesicatoria* 85-10, namely 10-methylundecan-2-one (**34**), has been reported from plants [43–46] and bacteria, e.g., from the myxobacterium *Stigmatella aurantiaca* and arctic bacteria of the *cytophaga-flavobacterium-bacteroides* group [47–48]. In some of these bacteria a pattern of methylketones similar to that of *X. c.* pv. *vesicatoria* 85-10 was found: A continuous row of homologous straight-chain methylketones accompanied by *iso*-branched isomers, showing 10-methylundecan-2-one (**34**) as a major component. To the best of our knowledge, the isomeric 9-methylundecan-2-one (**35**) has not been identified from a natural source before.

#### Possible biosynthesis of methylketones found in *Xanthomonas campestris* pv. *vesicatoria* 85-10

While odd-numbered, unbranched methylketones are clearly biosynthesized from straight-chain even-numbered fatty acids originating from the acetate pool, the biosynthesis of even-numbered congeners may start from propionate, a C3-unit. Consequently, odd-numbered fatty acids will yield even-numbered straight-chain methylketones. Leucine or valine may serve as the starters for the biosyntheses of *iso*-branched compounds [47–49], which, in this case, is supported by the presence of three esters of 3-methylbutanol and one of 2-methylbutanol. Similarly, *iso*-leucine (its involvement in biosynthesis being supported by the presence of esters of 2-methylbutanol) will give rise to *anteiso*-branching. However, due to the general principles of chain elongation with acetate/malonate units, in *X. c.* pv. *vesicatoria* 85-10 the corresponding methylketones showed an odd number of carbons along the chain. Consequently, the *anteiso*-branched isomers of compounds **21**, **30**, **40**, and **48** (Table 1) were absent among the natural volatiles.

**Scheme 2:**
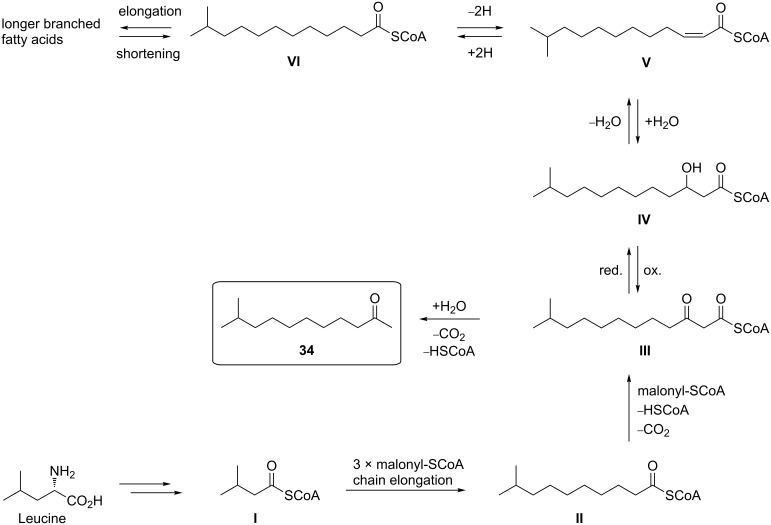
Suggested biosynthesis of methylketones found in *Xanthomonas campestris* pv. *vesicatoria* 85-10.

It is interesting to note that 10-methylundecan-2-one (**34**), the main component among the less volatile compounds emitted by *X. c.* pv. *vesicatoria* 85-10, shows a close biogenetic relation to 11-methyldodec-2*Z*-enoic acid (compound **V** in Scheme 2), which has been described earlier as a metabolite produced by *Xanthomonas* [13]. Possible biogenetic pathways to the two compounds are shown in Scheme 2. Chain elongation of 3-methylbutyryl-SCoA (**I**), produced from leucine, will give rise to the formation of (ω−1)-methylcarboxylic acids [47,50], and 10-methylundecan-2-one (**34**) may be either a degradation product of a longer branched-chain carboxylic acid or formed during an anabolic process. In any case, 11-methyl-3-oxodecanoyl-SCoA (**III**) will be the key compound in the formation of **34**. Starting from **I**, three complete cycles of chain elongation with malonyl-SCoA produces 9-methyldecanoyl-SCoA (**II**), which will yield **III** upon condensation with another malonyl-SCoA. After hydrolysis and decarboxylation, **III** will directly form **34**. On the other hand, reduction of **III** would furnish **IV**, and subsequent elimination of water would lead to **V**, the immediate precursor of 11-methyldodec-2*Z*-enoic acid. However, **V** may also be formed upon dehydrogenation of 11-methyldodecanoyl-SCoA (**VI**), which, in turn, may be produced by chain shortening of 13-methyltetradecanoyl-SCoA. Further steps during β-oxidation will afford **III** via **IV**.

With the exception of 8-methylnonan-2-one (**24**), an important component of the female-produced sex pheromone of the desert spider *Agelenopsis aperta* [51], no significant biological activities have yet been reported for the identified branched methylketones and methylcarbinols.

#### Various other volatiles are present in the profile of *X. c.* pv. *vesicatoria* 85-10

In addition to these methylketones and methylcarbinols, trace amounts of esters, such as 3-methylbutyl acetate (**6**) and 3-methylbutyl propionate (**14**), and the corresponding 2-methylbutyl esters **5** and **13** as well as 3-methylbutyl 3-methylbutyrate (**19**) could be identified. Trace amounts of benzylalcohol (**18**), acetophenone (**20**) and 2-phenylethanol (**23**) and its acetate (**29**) along with the three alkylated pyrazines **8**, **16**, **33** were the only aromatic components found in the bouquet, while geranylacetone (**39**) and a later-eluting yet unidentified compound, showing a mass spectrum somewhat resembling that of farnesylacetone, represented terpenoid structures among the volatiles produced by *X. c.* pv. *vesicatoria* 85-10.

#### PTR–MS of highly volatile compounds released by *X. c.* pv. *vesicatoria* 85-10

**Figure 4:**
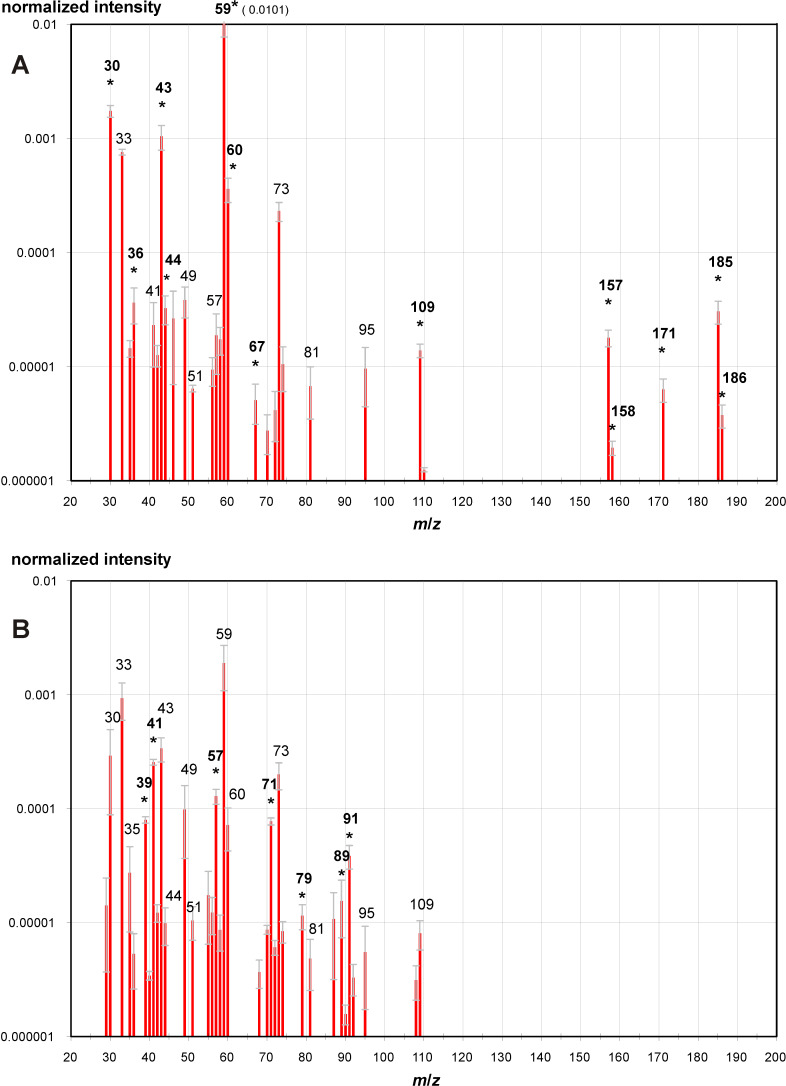
PTR–MS mass spectra of *Xanthomonas campestris* pv. *vesicatoria* 85-10 volatiles after three days of incubation. (A) PTR–MS profile scan of headspace volatiles of *X. c.* pv. *vesicatoria* 85-10 growing in a Petri dish with NB. (B) PTR–MS profile scan of headspace volatiles of *X. c.* pv. *vesicatoria* 85-10 growing in a Petri dish with NBG. Spectra are normalized to the primary ion signal (H_3_O^+^) and are blank corrected (i.e., spectra recorded from the respective media without bacteria are subtracted). Signals that are significantly influenced by the growth media (5% confidence level) are labeled by *. Only significant signals (mean subtracted by standard deviation) higher than 10^−6^ are shown and a log-scale on the ordinate axis is used. Note that missing signals therefore do not necessarily indicate zero concentration but may also be due to the variance of the sample or a blank signal higher than the mean value.

In our previous studies it became evident that the analysis of volatile profiles by GC/MS has its technical limitations for the detection of highly volatile compounds [34,52]. To extend the spectrum of volatiles of *X. c.* pv. *vesicatoria* 85-10, proton transfer reaction mass spectrometry (PTR–MS) was additionally used. The analyses of volatiles emitted by *X. c.* pv. *vesicatoria* 85-10 were performed after three days of incubation on NB and NBG in Petri dishes (Figure 4A and Figure 4B, respectively). At least 27 *m*/*z* values were found in the mass range between 30 and 250 with signals that were significantly enhanced (5% confidence level) compared to those of the blank control samples. If the proton affinity of an analyte molecule is higher than the one of H_3_O^+^ the proton transfer reaction is more or less quantitative (quasi-first-order reaction kinetics). Thus the detection efficiency of compounds is rather independent of the nature of the analyzed molecule [53] and the signal obtained by normalization of the measured count rate to the primary ion count rate is roughly proportional to the concentration. The headspace concentrations measured by online PTR–MS during the incubation experiments cover about four orders of magnitude. The most abundant *m*/*z* values (M + H)^+^ were 59, 30, 33, 41, 43, 57, 71, 73 and higher masses such as 157, 171 and 185 (Figure 4). As PTR–MS only provides molecular mass information, an unambiguous structure elucidation is impossible. However, the tentative assignments of some volatiles are strongly supported by the results obtained during GC/MS analysis (Figure 2, Table 1). The intensive signal at *m*/*z* 157 may be attributed to the sum of decan-2-one (**28**), 7-methylnonan-2-one (**25**) and 8-methylnonan-2-one (**24**); that at *m*/*z* 171 to the sum of undecan-2-one (**31**) and 9-methyldecan-2-one (**30**); and that at *m*/*z* 185 to the sum of dodecan-2-one (**38**), 9-methylundecan-2-one (**35**) and 10-methylundecan-2-one (**34**). As the instrument is tuned to maximum sensitivity, even the soft ionization by H_3_O^+^ could lead to some fragmentation due to the relatively high setting of the extraction/drift-tube voltages. Therefore, the *m*/*z* values of 41, 43, 57, 71, especially, may be fragments of short-chain alkylketones and aldehydes, as reported by Blake et al. in 2006 [54].

#### Changes in the compositions of headspace volatiles of *Xanthomonas campestris* pv. *vesicatoria* 85-10 during growth on different media

**Figure 5:**
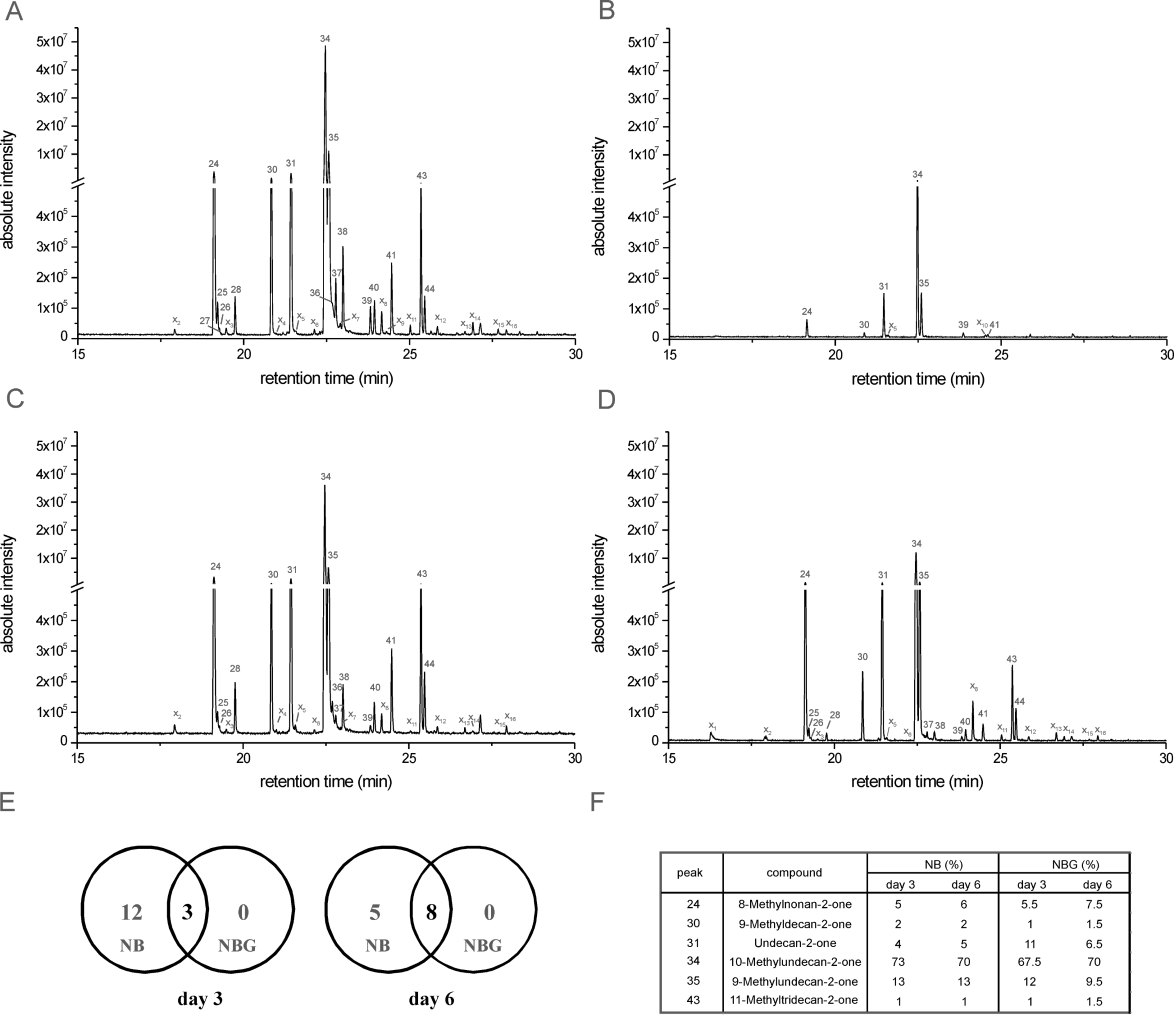
GC/MS analysis of volatiles emitted by *Xanthomonas campestris* pv. *vesicatoria* 85-10 grown on different media. (A) GC/MS-profile of headspace volatiles of *X. c.* pv. *vesicatoria* 85-10 grown on NB at day 3. (B) GC/MS-profile of headspace volatiles of *X. c.* pv. *vesicatoria* 85-10 grown on NBG at day 3. (C) GC/MS-profile of headspace volatiles of *X. c.* pv. *vesicatoria* 85-10 grown on NB at day 6. (D) GC/MS-profile of headspace volatiles of *X. c.* pv. *vesicatoria* 85-10 grown on NBG at day 6. (E) Registration of volatiles reaching a threshold level >1 × 10^5^ when *X. c.* pv. *vesicatoria* 85-10 grew on NB (left circle) and NBG (right circle) (day 3: left panel, day 6: right panel). (F) Relative contribution (%) of the six major volatiles emitted by *Xanthomonas campestris* pv. *vesicatoria* 85-10 at day 3 and 6 grown on NB and NBG.

Different intensities of reactions of the three tested fungi were observed when *X. c.* pv. *vesicatoria* 85-10 was either grown on NB or NBG (Figure 1). To trace this phenomenon, we applied solid-phase micro-extraction (SPME)–GC/MS as well as PTR–MS to monitor the patterns of headspace volatiles under two different growth conditions as well as at two different time points. The profiles of volatiles produced by *X. c.* pv. *vesicatoria* 85-10 growing on agar composed of NB and NBG were investigated at day 3 and day 6 after inoculation (Figure 5). A complex pattern of volatiles could be detected when *X. c.* pv. *vesicatoria* 85-10 was grown on the NB medium (Figure 5A). The pattern did not change much between day 3 and day 6 (Figure 5A and Figure 5C, Table 2). The volatile profile of *X. c.* pv. *vesicatoria* 85-10 grown on the NBG medium (day 3) proved to be simpler, both with respect to qualitative and quantitative composition, since only a few compounds could be traced (Figure 5B, Table 2). Interestingly, the number of detectable compounds increased after six days on NBG (Figure 5D, Table 2). By comparison of the bouquets released by *X. c.* pv. *vesicatoria* 85-10 growing on NB and NBG, only three overlapping compounds above a threshold level of 2 × 10^6^ were observed at day 3, whereas during continued incubation the compositions of bacterial volatiles became more alike, since eight compounds appeared in both chromatograms (Figure 5E, Table 2). This observation suggested a delay of volatile emission for *X. c.* pv. *vesicatoria* 85-10 grown on NBG. Two scenarios may explain this observation: (i) catabolite repression by glucose inhibited the synthesis of certain volatiles; or (ii) the easily accessible glucose was catabolized before other components of the medium were utilized.

**Table 2:** GC/MS analysis of SPME trapped volatiles emitted from *Xanthomonas campestris* pv. *vesicatoria* 85-10 grown in a Petri dish on NB or NBG.^a^

compound	identification	NB		NBG	
		day 3	day 6	day 3	day 6

**x_1_**	—	—	—	—	+
**x_2_**	—	+	+	—	+
**24**	8-methylnonan-2-one	+++	+++	+	+++
**25**	7-methylnonan-2-one	++	++	—	+
**26**	8-methylnonan-2-ol	+	+	—	+
**27**	7-methylnonan-2-ol	+	—	—	—
**x_3_**	—	+	+	—	+
**28**	decan-2-one	++	++	—	+
**30**	9-methyldecan-2-one	+++	+++	+	++
**x_4_**	—	+	+	—	—
**31**	undecan-2-one	+++	+++	++	+++
**x_5_**	—	+	+	+	+
**x_6_**	—	+	+	—	+
**34**	10-methylundecan-2-one	++++	++++	+	++++
**35**	9-methylundecan-2-one	++++	++++	++	+++
**36**	10-methylundecan-2-ol	++	+++	—	—
**37**	9-methylundecan-2-ol	++	+	—	+
**x_7_**	—	+	+	—	—
**38**	dodecan-2-one	++	++	—	+
**39**	geranylacetone	++	+	+	+
**40**	11-methyldodecan-2-one	++	++	—	+
**x_8_**	—	++	+	—	++
**x_9_**	—	+	—	—	—
**x_10_**	—	—	—	+	—
**41**	tridecan-2-one	++	++	+	+
**x_11_**	—	+	+	—	+
**43**	12-methyltridecan-2-one	++	++	—	++
**44**	11-methyltridecan-2-one	++	++	—	++
**x_12_**	—	+	+	—	+
**x_13_**	—	+	+	—	+
**x_14_**	—	+	+	—	+
**x_15_**	—	+	+	—	+
**x_16_**	—	+	+	—	+

^a^**x_1_**–**x_16_**: compounds not identified; + = 0–10^5^ counts; ++ = 10^5^–10^6^ counts; +++ = 10^6^–10^7^ counts; ++++ = 10^7^–10^8^ counts.

We also determined the contribution (%) of each of the six major volatiles during bacterial growth (Figure 5F). The dominant compound, 10-methylundecan-2-one (**34**) comprised about 70% under the four growth conditions, followed by 10–12% of 9-methylundecan-2-one (**35**), about 5–7% of 8-methylnonan-2-one (**24**), 4–6% undecan-2-one (**31**), and 1–2% 9-methyldecan-2-one (**30**) and 11-methyltridecan-2-one (**44**). All other compounds contributed less than 1% to the bouquets. It is interesting to note, that the ratios of the six major compounds did not significantly vary (except for undecan-2-one (**31**) at day 3 on NBG), indicating that the same metabolic pathways were active and only larger quantities/fluxes of the volatiles were emitted under the four different growth conditions.

Similarly, as performed with the GC/MS analysis, PTR–MS volatile profiles where obtained when *X. c.* pv. *vesicatoria* was grown on NB and NBG at day 3 and 6. The PTR–MS pattern of *X. c.* pv. *vesicatoria* grown on NB for three (Figure 4A) and six days were quite similar (data not shown). It is remarkable that, in comparison, the signals for the later-eluting alkanones, i.e., *m*/*z* 157, 171 and 185 especially, were absent or significantly lower when bacteria grew on NBG for three days (Figure 4B). Furthermore, *m*/*z* = 30, 36, 43, 44, 59, 60, 67 and 109 appeared at higher levels in NB compared to NBG. Conversely, when bacteria were grown on NBG, the signals at *m*/*z* = 39, 41, 57, 71, 79 as well as 89 and 91 were enhanced. When the incubation time on NBG was prolonged to six days, even the alkanones contributed significantly, but still in about five-fold lower amounts.

#### Individual volatiles of *Xanthomonas campestris* pv. *vesicatoria* 85-10 marginally influence fungal growth

**Figure 6:**
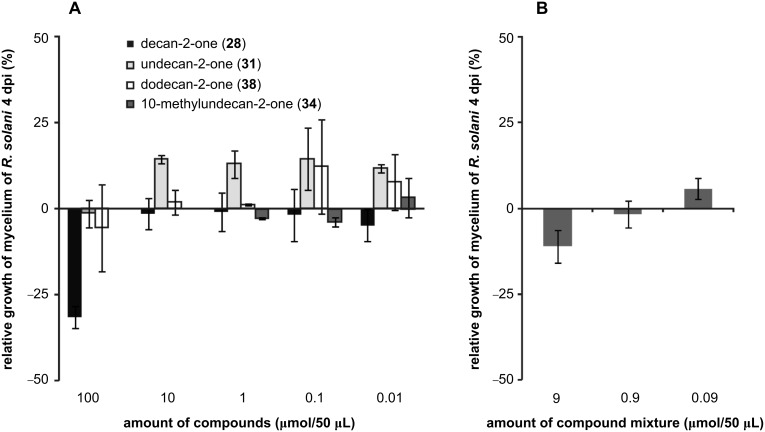
Testing synthetic volatiles on the growth of *Rhizoctonia solani.* Synthetic commercially available and chemically synthesized ketones were dissolved in pentane and applied on a filter-paper disc in aliquots of 50 μL. The filter paper was placed in the opposite compartment to the *R. solani* in a bi-partite Petri dish. Defined dilutions or mixtures were applied every 24 h. At day 4 the diameter of the mycelium was measured and compared to control plates containing pentane. Positive values represent growth promotion and negative values growth inhibition of *R. solani* compared to the control. Data are from two experiments each replicated three times; the standard deviation is presented. (A) Decan-2-one (**28**) (black), undecan-2-one (**31**) (light grey), dodecan-2-one (**38**) (white), and 10-methylundecan-2-one (**34**) (dark grey) were individually applied in 50 μL aliquots each day (day 0 to day 4). (B) A mixture of **28**, **31**, **38** and **34** (6.7%:5.5%:0.6%:87.2%) was applied in 50 μL aliquots every day (day 0 to day 4).

As demonstrated in Figure 1, *X. c.* pv. *vesicatoria* 85-10 strongly inhibited the growth of *Rhizoctonia solani* and *Aspergillus nidulans* and to a certain extent that of *Fusarium solani*. The inhibition was stronger when *X. c.* pv. *vesicatoria* 85-10 was grown on NB as compared to NBG. Both methods, GC/MS and PTR–MS, indicated the emission of a multitude of volatile compounds, including many ketones. Decan-2-one (**28**), undecan-2-one (**31**), dodecan-2-one (**38**) and 10-methylundecan-2-one (**34**) were identified as major products of *X. c.* pv. *vesicatoria* 85-10. Since the ketones were released in different quantities from both media, we tested these ketones individually in different amounts or in combination, to find out whether the identified ketones influence the fungal growth (Figure 6). They were applied repetitively every 24 h at concentrations of 0.01, 0.1, 1.0, 10 and 100 μmol in 50 μL pentane (10-methylundecan-2-one (**34**) was applied at 0.01, 0.1 and 1 μmol in 50 μL pentane). The area of the fungal mycelium was determined after four days of cocultivation. Only at a concentration of 100 μmol decan-2-one (**28**) the growth of *R. solani* was inhibited, by 30% (Figure 6), while the other ketones did not significantly influence the development of *R. solani* at any of the tested concentrations. Surprisingly, undecan-2-one (**31**) promoted the growth of *R. solani* by 10–15% at concentrations from 0.01 to 10 μmol in 50 μL and dodecan-2-one (**38**) at 0.01 and 0.1 μmol in 50 μL. 10-Methylundecan-2-one (**34**) had no effect on the growth of *R. solani*. An additional bioassay, performed with a mixture of the four synthetic compounds (decan-2-one (**28**), undecan-2-one (**31**), dodecan-2-one (**38**), 10-methylundecan-2-one (**34**)) in the ratios that were emitted by the bacteria growing in the Petri dish (6.7%:5.5%:87.2%:0.6%), revealed 10% inhibition at 9 μmol in 50 μL and slight promotion (ca. 6%) at 0.09 μmol in 50 μL. These results indicate that the individual ketones neither acted additively nor synergistically, and they were most likely not significantly responsible for inhibitory effects on the growth (Figure 1) of the fungi by *X. c.* pv. *vesicatoria* volatiles. We concluded that either the experimental design of the bioassay was not appropriate or other volatiles account for the inhibitory effects. In the literature, contradictory results were found for undecan-2-one (**31**). It is likely to be involved in the inhibition of sapstain fungi [40], while *Sclerotinia sclerotiorum* was not affected by **31** [22]. It must also be considered that it is not known whether the emitted volatile blends change qualitatively and quantitatively during the growth of *X. c.* pv. *vesicatoria* 85-10 [34]. Hence, to find the inhibitory component(s) and the bioactive concentrations, the emitted blends of volatiles must be investigated more comprehensively.

In addition, inorganic compounds have to be considered, including hydrogen cyanide (HCN) and ammonia. HCN is produced, e.g., by *Pseudomonas* spp. [55] and, due to its slightly higher proton affinity than water, it should be detectable down to a concentration lower than 100 ppb by PTR–MS [56–57]. Nevertheless in our experiments with *X. c.* pv. *vesicatoria* 85-10 we did not detect any significant signal at *m*/*z* = 28 corresponding to HCN. Ammonia emissions by *X. vesicatoria* and of two *Bacillus subtilis* strains in cocultivation with *Neurospora crassa* were described [58–59]. Ammonia is of course an important nitrogen source for organisms, but recently other roles were also attributed to ammonia. Nijland and Burgess [60] suggested that ammonia acts as an olfactory cue in the communication of *Bacillus licheniformis* strains, and Bernier et al. [61] showed that elevated ammonia levels lead to increased polyamine levels in *E. coli* with consequences for the membrane permeability and increased antibiotic resistance. Other volatiles such as CO, CH_4_, CH_2_O and NO were not detectable with the applied methods. However, the PTR provides evidence for the emission of sulfur-containing compounds such as H_2_S (*m*/*z* = 35) and methanethiol (*m*/*z* = 49), as has been suggested for other microorganisms [52]. The compound producing *m*/*z* = 33 was tentatively assigned to be methanol in accordance with published data [52]. Methanol is so far not known as a prominent compound released by bacteria. In addition to the *Enterobacteriaceae E.coli, Shigella flexneri*, and *Salmonella enterica* also *X. vesicatoria* may emit methanol*.* The release of CO_2_ due to the active tricarboxylic acid cycle is very likely, but CO_2_ does not accumulate at higher than ambient concentrations in the open experimental system [62] and, therefore, cannot be considered as a growth-promoting or -inhibitory component.

### Considerations concerning methodological approaches

It is quite obvious that further research is necessary to identify the bioactive compounds, especially those sensitive and/or highly volatile substances that escaped conventional GC/MS-analysis. During our investigations on volatiles of *Xanthomonas* we noticed a discrepancy in the number of compounds detected in the headspace of a 10 L liquid-medium culture versus growth on solid medium (Petri dish). Beside the known physiological adaptations of bacteria growing on liquid and solid media, the different trapping material (Super-Q versus polydimethylsiloxane) and the experimental procedure (30 hour trapping versus 2 hour trapping) most likely account for those differences. As discussed above, the suitability of PTR–MS for compound identification is limited. Therefore, structure assignments are based on comparison with GC/MS data (i.e., as performed also in this work) and literature-based information. Selectivity may be enhanced by using ammonia as the primary ion-source gas. Ammonia has a higher proton affinity than water, and therefore, some substance classes can be ionized selectively (e.g., nitrogen-containing compounds). Further information to discriminate nominally isobaric compounds could be obtained by using proton-transfer-reaction ion-trap mass spectrometry (PIT–MS), which allows tandem mass spectrometric experiments (MS/MS) [63–64], or by high-resolution PTR–TOF–MS for the determination of exact molecular masses and assignment of atomic compositions. Regarding quantitative analysis, some striking differences between results obtained with extracts or solid-phase micro extraction (SPME) applying conventional GC/MS and PTR–MS, respectively, have been observed. As an example, in PTR–MS analyses the signal at *m*/*z* = 109 is about as intensive as those produced by the methyl ketones at *m*/*z* = 157, *m*/*z* = 171, and *m*/*z* = 185 (Figure 4A). In contrast, in our GC/MS analyses the sum of 2,5-dimethylpyrazine (**8**) and benzylalcohol (**18**) (Figure 2, Table 1), which may give rise to *m*/*z* = 109 (provided that there is no abundant compound that did not survive GC), never exceeded 5% of 10-methylundecan-2-one (**34**). These discrepancies require further investigations. The most important future analytical aspect will be online PTR–MS, tracing the dynamics in the emission of volatiles during incubation time, considering both qualitative and quantitative compositions of the profiles. In addition, gas-chromatographic analyses of highly volatile compounds, by using either direct headspace injection or solid-phase adsorption/thermodesorption separation on thick film capillaries connected to a GC/MS system, will have to be carried out.

## Conclusion

Profiles of volatiles emitted by *Xanthomonas c.* pv. *vesicatoria* 85-10 were investigated by using GC/MS and PTR–MS techniques. More than 50 compounds were emitted by this species, the majority comprising ketones and methylketones, including 10-methylundecan-2-one (**34**) as the largely dominant component. The emission profiles differed depending on whether the bacteria were grown on a medium with or without glucose. To better understand the involvement of *X. c.* pv. *vesicatoria* volatiles in antagonistic processes against fungi, more ecological information is required. Most obviously it needs to be addressed as to whether *X. c.* pv. *vesicatoria* 85-10 emits volatiles while growing in the phyllosphere and whether the emission occurs continuously. Continuous volatile perception by the plant may be a prerequisite to induce signal cascades in the plant, similarly to the behavior shown for repetitive wounding in contrast to single cuts in plants [65]. Furthermore, a possible role of volatiles in achieving a better colonization of the aerial parts by *X. c.* pv. *vesicatoria* has to be considered. Another question of concern is whether and how the plants are directly affected by the volatiles of *X. c.* pv. *vesicatoria* 85-10. Plant growth promotion as well as inhibition is reflected at the molecular and physiological level of *Arabidopsis thaliana* and of *Physcomitrella patens* [23,27,31,34,62,66]. However, in most of these studies the bioactive compound(s) need to be determined. Therefore, future tasks must focus on (i) the comprehensive determination of volatile profiles of bacteria of the phyllosphere as well as in the rhizosphere and (ii) the identification of biologically active volatiles.

## Experimental

### Bacterial cultures

*Xanthomonas campestris* pv. *vesicatoria* 85-10 was originally isolated from *Capsicum* sp. The bacterial strain was kindly provided by Prof. U. Bonas (University of Halle/Wittenberg). It was stored in frozen stocks at −70 °C and grown on nutrient broth (NB) II agar (SIFIN, Berlin) either with or without 1.1% glucose (Merck, Darmstadt) at 30 °C.

### Fungal isolates

*Aspergillus nidulans* (FGCS A4) was kindly provided by Dr. S. Busch (University of Göttingen). *Fusarium solani* and *Rhizoctonia solani* KÜHN (RHI S0 WE) were obtained from the Strain Collection of Antagonistic Microorganisms (SCAM; University of Rostock, Microbiology). Fungal mycelium was grown on Sabouraud agar (Dinkelberg Analytics GmbH, Regensburg) with a mycelia plug from stock cultures stored at −70 °C. To maintain the fungi, every seventh day a mycelium disk was transferred from the edge of an actively growing fungus to a new agar plate. Cultures were incubated at 20 °C in the dark.

### Cocultivation of *Xanthomonas campestris* pv. *vesicatoria* 85-10 with fungi

Bacteria were grown on nutrient agar (NB II; peptone from casein 3.5 g·L^−1^, peptone from meat 2.5 g·L^−1^, peptone from gelatin 2.5 g·L^−1^, yeast extract 1.5 g·L^−1^, NaCl 5 g·L^−1^, agar-agar 15 g·L^−1^, pH 7.2), and nutrient agar with glucose (NBG; NB plus 11 g·L^−1^ glucose) at 30 °C. Cocultivations of bacteria and fungi were performed in bi-partite Petri dishes. One milliliter of the overnight cultures of *Xanthomonas* grown on NB or NBG was transferred into 100 mL fresh medium in 250 mL chicane flasks and incubated by shaking overnight at 30 °C. Fifty microliters of these cultures were plated onto nutrient agar, with or without glucose, in one compartment of the Petri dish and cultivated at 30 °C. Three days later (day 0 of cocultivation) a seven-day-old 6 mm disk of actively grown mycelium of the tested fungus was transferred to Sabouraud agar in the other compartment of the Petri dish. Cocultivation was performed at 20 °C in the dark. Digital images were taken after four days. Growth of fungi was determined by measuring the diameter of the mycelium and compared to the control (without adjacent bacterial growth). Average values were calculated based on three experiments each with three to five replicates.

### Analysis of volatiles produced by *Xanthomonas campestris* pv. *vesicatoria* 85-10

**Cultivation in liquid medium:** A single colony of *X. c.* pv. *vesicatoria* was picked and suspended in 6 mL of NB medium. After incubation for 24 h at 30 °C and shaking at 180 rpm, 1 mL (OD_600_ = 0.8–1.4) was used as inoculum for a 250 mL NB culture. The preculture (100 mL) was used to inoculate 10 L of the culture medium in a 20 L Duran® wide-necked glass flask closed with a specially designed glass cap (equipped with an inlet and an outlet). During growth, the bacterial culture was stirred with a magnetic stir bar at 600 rpm and incubated at 30 °C. A diaphragm pump (Denver Gardner, Puchheim, Germany) pulled charcoal-purified, sterile air at a flow of 3 L/min through the system. Volatiles released during a 30 hours growth of *X. c.* pv. *vesicatoria* were trapped on Super-Q (100 mg, Alltech Associates, Deerfield, IL, USA). The collected volatiles were eluted three times with 500 μL dichloromethane and further analysed. Gas chromatography coupled with mass spectrometry (GC/MS) was carried out by linking a gas chromatograph HP 5890 (Hewlett Packard, Palo Alto, US) to a double-focusing spectrometer (VG-7070/250SE, Vacuum Generators, Manchester, UK). By using helium as the carrier gas, separations were achieved with a 50 m × 0.25 mm id CPSil5 fused silica column (Chrompack, Middleburg, The Netherlands) under the following conditions: injector temperature 250 °C, 1 min splitless, 1 min at 50 °C, then programmed to increase to 290 °C at a rate of 3.5 °C/min. Identification of volatiles was based on comparison of their mass spectra with those reported in the literature [67–68] as well as with the analytical data of commercially available or specifically synthesized reference compounds (co-injection). Synthetic 10-methylundecan-2-one and 9-methylundecan-2-one served as standards for the structure assignments of other branched-chain methyl ketones on the basis of their mass spectra and retention indices.

**Cultivation on solid medium:** In another analytical approach, solid-phase micro extraction (SPME) was applied to trap volatiles, which were subsequently analyzed by GC/MS. Bi-partite glass Petri dishes with a borehole (1 mm diameter) at the side of one compartment were used for SPME. The preculture (1 mL) was used to inoculate 100 mL NB or NBG medium in a 250 mL chicane flask. After 24 h of incubation at 30 °C, 50 μL was plated on NB or NBG agar on one side of the compartmentalized Petri dish. Similarly to the cocultivation tests, the inoculated plate was incubated for three days at 30 °C under darkness. The collection of volatiles was performed at day 3 and day 6 after inoculation at 20 °C in the dark. A 100 μm polydimethylsiloxane (PDMS; SUPELCO, Bellefonte, USA) coated SPME-fiber was preconditioned to remove all contaminants (30 min, 250 °C). Subsequently, the SPME needle was introduced into the Petri dish through the hole, and the fiber was extended into the headspace. The time for the adsorption and accumulation of bacterial volatiles was set to 2 h. Volatile compounds were thermally desorbed and analyzed by using a GC/MS-QP5000 (Shimadzu; Kyoto, Japan, injection port set at 250 °C). The initial temperature of the DB5-MS column (60 m × 0.25 mm × 0.25 μm; J&W Scientific, Folsom, California, USA) was kept at 35 °C for 5 min, increased to 280 °C at a rate of 10 °C/min, and then kept constant for 15 min. Helium at a flow rate of 1.1 mL/min was used as the carrier gas with a linear velocity of 28 cm/s. Electron ionization (EI) mass spectra were taken at 70 eV. The mass range was *m*/*z* = 40–280. Compounds were identified by using available reference compounds and the library of the National Institute of Standards and Technology (NIST147) for the comparison of mass spectra, retention times and Kovats indices [69]. Analysis of the volatile emissions was replicated twice, and compounds emitted by the NB or NBG agar (blank) were subtracted.

Highly volatile components produced by *X. campestris* pv. *vesicatoria* 85-10 were monitored by proton transfer reaction mass spectrometry (PTR–MS). This technique allows continuous online monitoring of mixtures of volatiles at the parts-per-billion (ppb) level. The method is a special variant of the well-established chemical ionization mass spectrometry. In PTR–MS, the hydronium ion H_3_O^+^ is the primary species to generate the protonated analyte (M + H)^+^, which is recorded by the detector. The PTR–MS (Ionicon, Innsbruck, Austria) used in this investigation has been described elsewhere [70]. As a modification to this original PTR–MS a heated inlet system was installed to prevent condensation and to enable also a qualitative analysis of less volatile compounds. It consists of a deactivated 2 m long GC capillary (ID 0.53 mm, MXT Guard Column, BGB-Analytik, Schlossboeckelheim, Germany) integrated in a 1/8 inch copper tube and maintained at a temperature of 60 °C by using a heating hose (Horst GmbH, Lorsch, Germany). The sample flow through the capillary is 80 mL/min. Drift tube pressure was set to 2.0 mbar and drift voltage to 600 V. These settings resulted in very sensitive measurements, but some side reactions led to fragmentation of, e.g., aldehydes, as described for a SWIFT instrument by Blake et al. [54]. Mass-to-charge ratios from 20 to 250 unified mass units (*m*/*z*) were run by using a quadrupole at a dwell time of 100 ms per *m*/*z* resulting in a repetition rate of about two full mass spectra per minute. Generally 100 spectra were recorded. For PTR-analysis the Petri dish with growing bacteria were placed at day 3 and 6, without the lid, into a glass compartment (Petri dish, 145 x 30 mm) with an in- and outlet. Charcoal-purified air was passed over the culture and entered the PTR instrument through the inlet. All analyses of bacterial metabolites were carried out in triplicate. Blank samples of NB and NBG were analyzed twice to identify compounds directly related to outgasing of the nutrient agar. For data evaluation, raw spectra of each measurement (100 spectra) were first normalized to the signal of the primary ion (H_3_O^+^), to enable a semiquantitative data evaluation and a direct comparison of spectra that were obtained at different days with a slightly different sensitivity. As a second step these normalized raw spectra were averaged and blank-value corrected. Statistical analyses of bacterial metabolites on NB and NBG were performed by using a variant of the T-test, with a 5% confidence level [71].

### Syntheses of reference compounds

The syntheses of 10-methylundecan-2-one (**34**) and 9-methylundecan-2-one (**35**) proceeded straightforwardly, following a conventional alkyne approach (Scheme 1). Commercially available (Aldrich) 4-pentyne-2-ol was reacted with benzyl chloride to yield the corresponding benzyl ether **A**. Subsequently, **A** was coupled to commercially available (Aldrich) 1-bromo-5-methylhexane (**B**) according to the standard procedure [72]. The resulting 2-benzyloxy-10-methylundec-4-yne (**C**) was hydrogenated over 10% Pd/C-catalyst at 1 atm. The crude secondary alcohol was oxidized with pyridinium dichromate [73–74] to yield the corresponding ketone. Purification by column chromatography on silica (ICN, pore size 60 Å, particle size 32–63 μm) with cyclohexane/ethyl acetate 20:1 followed by Kugelrohr distillation at 20 mmHg afforded 10-methylundecan-2-one (**34**) in a purity of 98%. Analytical data of the product were identical to those reported in the literature [47]. Following the same route as for the synthesis of **34** (Scheme 1), 9-methylundecan-2-one (**35**), was prepared by coupling 1-bromo-4-methylhexane (**B′**) [75] to **A**. Hydrogenation of the obtained **C′** followed by oxidation of the obtained alcohol yielded **35**.

**Synthesis of 9-methylundecan-2-one (35):** To a stirred, ice-cooled solution of 2.0 g (23.8 mmol) *rac*-4-pentyne-2-ol in 50 mL dry THF was slowly added 1.20 g (49.9 mmol) sodium hydride. The reaction mixture was warmed to room temperature, and stirring was continued until the formation of hydrogen ceased. Subsequently, a solution of 4.27 g (25.0 mmol) benzyl bromide in 50 mL dry THF was added slowly. After 3 h stirring at RT, 150 mL water was added, and the mixture was extracted with three portions of diethyl ether at 50 mL each. The combined organic solutions were washed with a saturated aqueous solution of sodium hydrogen carbonate and brine and dried over magnesium sulfate. The solvent was removed in vacuo, and the residue was submitted to flash chromatography (silica; cyclohexane/ethyl acetate 40:1) yielding 2.44 g (29.8 mmol, 83%) of 2-benzyloxypent-3-yne (**A**).

A solution of 2.70 g (15.5 mmol) of **A** in 50 mL dry THF was cooled to −78 °C and deprotonated with 10.6 mL of a 1.6 M solution of butyl lithium in hexane, which was added slowly. After warming to RT, 3.05 g (17.0 mmol) of 1-bromo-4-methylhexane (**B’**), dissolved in 15 mL dry THF, was added dropwise. After heating under reflux for 12 h, the mixture was cooled to RT, and 150 mL of a saturated aqueous solution of ammonium chloride was added. After separation, the aqueous layer was extracted with three portions of diethyl ether at 50 mL each. The combined organic solutions were washed with brine and dried over magnesium sulfate. The solvent was removed in vacuo, and the residue was submitted to flash chromatography (silica; cyclohexane/ethyl acetate 40:1) to yield 2.7 g (10.0 mmol, 64%) of 2-benzyloxy-9-methylundec-4-yne (**C′**). A solution of 2.53 g (9.3 mmol) of crude **C′** in 50 mL methanol was hydrogenated overnight at 20 bar by using 20% PD/C-catalyst. The mixture was filtered over silica, and the solvent was removed in vacuo.

To a solution of 950 mg (5.1 mmol) of the crude 9-methylundecane-2-ol in 50 mL dichloromethane was added 2.3 g (1.2 equiv) pyridinium dichromate, and the mixture was vigorously stirred for 12 h at RT. As a gas-chromatographic control revealed the reaction to be incomplete, another 1.2 equiv of pyridinium dichromate was added, and the mixture was heated under reflux for 6 h. After filtration over silica and concentration in vacuo, the crude ketone was purified by column chromatography on silica with cyclohexane/diethyl ether 20:1. A final Kugelrohr distillation of 20 mmHg afforded 855 mg (4.6 mmol) of 9-methylundecan-2-one (**35**). The total yield over four steps was about 45%, the obtained product showing a purity of ca. 98%. ^1^H NMR (500 MHz, CDCl_3_) δ 0.83 (d, *J* = 6.1 Hz, 3H, CH_3_ C12), 0.85 (t, *J* = 7.2 Hz, 3H, CH_3_ C11), 1.03–1.10/1.22–1.30 (2m, 2H, CH_2_ C8), 1.06–1.17/1.20–1.30 (2m, 2H, CH_2_ C10), 1.21–1.32 (m, 6H, 3×CH_2_ C5-C7), 1.24–1.33 (m, 1H, CH C9), 1.52–1.61 (m, 2H, CH_2_ C4), 2.13 (s, 3H, CH_3_ C1), 2.41 (t, *J* = 7.5 Hz, 2H, CH_2_ C3) ppm; ^13^C NMR (101 MHz, C_6_D_6_) δ 11.54 (q, C11), 19.35 (q, C12), 24.04 (t, C4), 27.06 (t, C7), 29.38 (t, C5), 29.62 (t, C10), 29.90 (t, C6), 29.99 (q, C1), 34.52 (d, C9), 36.70 (t, C8), 43.99 (t, C3), 209.16 (s, C2) ppm; GC/MS (EI, 70 eV) *m*/*z* (% relative intensity): 39 (8), 41 (35), 42 (6), 43 (100), 56 (7), 57 (23), 58 (94), 59 (37), 67 (4), 68 (3), 69 (9), 70 (11), 71 (49), 81 (6), 82 (8), 83 (6), 85 (11), 95 (9), 96 (14), 97 (8), 109 (5), 110 (3), 124 (4), 126 (4), 127 (4), 137 (4), 166 (2), 169 (1), 184 (4); Anal. calcd for C_12_H_24_O: C, 78.20; H, 13.12; found: C, 77.52; H, 13.16.

### Influence of individual volatiles of *Xanthomonas campestris* pv. *vesicatoria* 85-10 on the growth of different fungi

Synthetic ketones identified as volatiles of *Xanthomonas* (decan-2-one (**28**), undecan-2-one (**31**), 10-methylundecan-2-one (**34**), dodecan-2-one (**38**)) were assayed in bi-partite Petri dishes with *R. solani* as the test organism*.* A seven-day-old 6 mm disk of actively grown mycelium was transferred to Sabouraud agar, to one compartment of the Petri dish. Pentane solutions of synthetic compounds were prepared in different concentrations in decade steps (0.01–100 μmol in 50 μL). In addition, a blend of the four synthetic compounds in defined ratios (**28**:**31**:**34**:**38** 6.7%:5.5%:87.2%:0.6%) was tested as well. Fifty microliters of each mixture (0.09–9 μmol) were applied on filter paper (1 cm^2^), which was deposited at the other compartment of the Petri dish. Control experiments were performed with pentane alone. Similarly to the cocultivation test, the bioassay with individual compounds was performed at 20 °C in the dark. Every 24 h the filter paper was replaced by a freshly prepared filter paper with 50 μL of the respective test solution. Growth inhibition of fungi was determined by measuring the diameter of the mycelium at day 4 of the experiment and by comparison to the control experiment (pure pentane). Average values were calculated based on two repeat experiments each with three replicates.

## References

[1] Ruinen J (1961). Plant Soil.

[2] Last F T, Deighton F C (1965). Trans Br Mycol Soc.

[3] Blakeman J P, Fokkema N J (1982). Annu Rev Phytopathol.

[4] Preece T F, Dickinson C H (1971). Ecology of leaf surface microorganisms.

[5] Hirano S S, Upper C D (2000). Microbiol Mol Biol Rev.

[6] Yang C H, Crowley D E, Borneman J, Keen N T (2001). Proc Natl Acad Sci U S A.

[7] Lindow S E, Leveau J H J (2002). Curr Opin Biotechnol.

[8] Lindow S E, Brandl M T (2003). Appl Environ Microbiol.

[9] Doidge E M J (1921). Dep Agric, Union S Afr.

[10] Hayward A C, Swings J G, Civerolo E L (1993). The hosts of Xanthomonas. Xanthomonas.

[11] Gürlebeck D, Thieme F, Bonas U (2006). J Plant Physiol.

[12] Opelt K, Berg G (2004). Appl Environ Microbiol.

[13] Wang L, He Y, Gao Y, Wu J E, Dong Y, He C, Wang S X, Weng L, Xu J, Tay L (2004). Mol Microbiol.

[14] Hogan D A, Vik A, Kolter R (2004). Mol Microbiol.

[15] Rasmann S, Köllner T G, Degenhardt J, Hiltpold I, Toepfer S, Kuhlmann U, Gershenzon J, Turlings T C J (2005). Nature.

[16] Hamilton-Kemp T R, McCracken C T, Loughrin J H, Andersen R A, Hildebrand D F (1992). J Chem Ecol.

[17] Pandey V N, Dubey N K (1992). Biol Plant.

[18] Trombetta D, Castelli F, Sarpietro M G, Venuti V, Cristani M, Daniele C, Saija A, Mazzanti G, Bisignano G (2005). Antimicrob Agents Chemother.

[19] Wink M, Carpinella M C, Rai M (2006). Importance of plant secondary metabolites for protection against insects and microbial infections. Naturally occurring bioactive compounds.

[20] Kai M, Haustein M, Molina F, Petri A, Scholz B, Piechulla B (2009). Appl Microbiol Biotechnol.

[21] Wenke K, Kai M, Piechulla B (2010). Planta.

[22] Fernando W G D, Ramarathnam R, Krishnamoorthy A S, Savchuk S C (2005). Soil Biol Biochem.

[23] Kai M, Effmert U, Berg G, Piechulla B (2007). Arch Microbiol.

[24] Schulz S, Dickschat J S (2007). Nat Prod Rep.

[25] Kai M, Piechulla B (2010). Plant Signal Behav.

[26] Fiddaman P J, Rossall S (1993). J Appl Microbiol.

[27] Vespermann A, Kai M, Piechulla B (2007). Appl Environ Microbiol.

[28] Chun W, Cui J, Poplawsky A (1997). Physiol Mol Plant Pathol.

[29] Fiddaman P J, Rossall S J (1994). Appl Bacteriol.

[30] Strobel G A, Dirkse E, Sears J, Markworth C (2001). Microbiology (Reading, U K).

[31] Ryu C M, Farag M A, Hu C H, Reddy M S, Wie H X, Pare P W, Kloepper J W (2003). Proc Natl Acad Sci U S A.

[32] Zhang H, Kim M S, Krishnamachari V, Payton P, Sun Y, Grimson M, Farag M A, Ryu C M, Allen R, Melo I S (2007). Planta.

[33] Zhang H, Xie X, Kim M S, Kornyeyev D A, Holaday S, Pare P W (2008). Plant J.

[34] Kai M, Crespo E, Cristescu S M, Harren F J M, Francke W, Piechulla B (2010). Appl Microbiol Biotechnol.

[35] Blom D, Fabbri C, Eberl L, Weisskopf L (2011). Appl Environ Microbiol.

[36] Wenke K, Weise T, Warnke R, Valverde C, Wanke D, Kai M, Piechulla B, Witzany G, Baluska F (2012). Bacterial Volatiles Mediating Information Between Bacteria and Plants. Bio-Communication in Plants.

[37] Ryan R P, Dow J M (2008). Microbiology (Reading, U K).

[38] Katritzky A R, Chen K (2000). Anal Chem.

[39] Schulz S (2001). Lipids.

[40] Junkes B S, Amboni R D M C, Heinzen V E F, Yunes R A (2002). Chromatographia.

[41] Bruce A, Verrall S, Hackett C A, Wheatley R E (2004). Holzforschung.

[42] Höckelmann C, Moens T, Jüttner F (2004). Limnol Oceanogr.

[43] Tressl R, Friese L Z (1978). Lebensm-Unters Forsch.

[44] Joulain D, Laurent R, Fourniol Y P, Yaacob K B J (1991). Essent Oil Res.

[45] Schlumpberger B O, Clery R A, Barthlott W (2006). Plant Biol.

[46] Altintas A, Koca U, Demirci B, Husnu Can Baser K (2010). Asian J Chem.

[47] Dickschat J S, Bode H B, Wenzel S C, Müller R, Schulz S (2005). ChemBioChem.

[48] Dickschat J S, Helmke E, Schulz S (2005). Chem Biodiversity.

[49] Dickschat J S, Wenzel S C, Bode H B, Müller R, Schulz S (2004). ChemBioChem.

[50] Francke W, Schulz S, Barton D, Nakanishi K (1999). Pheromones. Miscellaneous Natural Products Including Marine Natural Products, Pheromones, Plant Hormones, and Aspects of Ecology.

[51] Papke M D, Riechert S E, Schulz S (2001). Anim Behav.

[52] Bunge M, Araghipour N, Mikoviny T, Dunkl J, Schnitzhofer R, Hansel A, Schinner F, Wisthaler A, Margesin R, Märk T D (2008). Appl Environ Microbiol.

[53] Lindinger W, Hansel A, Jordan A (1998). Int J Mass Spectrom Ion Processes.

[54] Blake R S, Wyche K P, Ellis A M, Monks P S (2006). Int J Mass Spectrom.

[55] Knowles C J (1976). Bacteriol Rev.

[56] Hunter E P L, Lias S G (1998). J Phys Chem Ref Data.

[57] Knighton W B, Fortner E C, Midey A J, Viggiano A A, Herndon S C, Wood E C, Kolb C E (2009). Int J Mass Spectrom.

[58] Ryan F J, Schneider L K (1947). J Bacteriol.

[59] Stall R E, Hall C B, Cook A A (1972). Phytopathology.

[60] Nijland R, Burgess J G (2010). Biotechnol J.

[61] Bernier S P, Létoffé S, Delepierre M, Ghigo J M (2011). Mol Microbiol.

[62] Kai M, Piechulla B (2009). FEBS Lett.

[63] Steeghs M M L, Crespo E, Harren F J M (2007). Int J Mass Spectrom.

[64] Crespo E, Cristescu S M, de Ronde H, Kuijper S, Kolk A H J, Anthony R M, Harren F J M (2011). J Microbiol Methods.

[65] Bricci I, Leitner M, Foti M, Mithöfer A, Boland W, Maffei M E (2010). Planta.

[66] Kai M, Vespermann A, Piechulla B (2008). Plant Signal Behav.

[67] McLafferty F W, Stauffer D B (1989). The Wiley/NBS Registry of Mass Spectral Data.

[68] Adams R P (2007). Identification of Essential Oil Components by Gas Chromatography/Mass Spectometry.

[69] Kováts E sz (1961). Fresenius' Z Anal Chem.

[70] Hansel A, Jordan A, Holzinger R, Prazeller P, Vogel W, Lindinger W (1995). Int J Mass Spectrom Ion Processes.

[71] de V. Weir J B (1960). Nature.

[72] Brandsma L (1988). Preparative Acetylenic Chemistry.

[73] Corey E J, Schmidt G (1979). Tetrahedron Lett.

[74] Cernecki S, Georgoulis C, Stevens C L, Vijayakumaran K (1985). Tetrahedron Lett.

[75] Sonnet P E, Carney R L, Henrick C (1985). J Chem Ecol.

